# Global volcanic rock classification of Holocene volcanoes

**DOI:** 10.1038/s41597-023-02324-7

**Published:** 2023-07-01

**Authors:** Frédérique Oggier, Christina Widiwijayanti, Fidel Costa

**Affiliations:** 1 School of Physical and Mathematical Sciences, Nanyang Technological University, Singapore, Singapore; 2 Earth Observatory of Singapore, Nanyang Technological University, Singapore, Singapore; 3 Asian School of the Environment, Nanyang Technological University, Singapore, Singapore; 4 Institut de Physique du Globe de Paris, Université Paris Cité, CNRS, Paris, France

**Keywords:** Natural hazards, Volcanology

## Abstract

This data descriptor assigns the major and minor rock names from worldwide Holocene volcanoes of the Global Volcanism Program (GVP) using the Total Alkali-Silica diagram (TAS) for the chemical classification of volcanic rocks using the Geochemistry of Rocks of the Oceans and Continents (GEOROC) database. The precompiled files of the GEOROC database provide the chemical composition of volcanic rock samples, from which we computed major and minor rocks for global Holocene volcanoes reported in GVP. The combined dataset associates each volcano with the relative abundance of each volcanic sample type (whole rock, glass, melt inclusion) and provides the five major (more than 10% abundance) and minor rock names. In total, over 138,000 GEOROC volcanic rock samples were considered, for ~1000 Holocene volcanoes. The resulting major rock compositions are in general consistent with those given in GVP. The dataset provides a global panorama of rock composition for Holocene volcanoes.

## Background & Summary

Volcanic rocks are a record of magmatic processes, from the source origin of the magma, its residency in the crustal plumbing system, and its eventual eruption at the earth’s surface^1,2^. These processes are reflected in the petrological and geochemical composition of the rocks, which reveal changes in magma storage and transport^3,4^ and are a key factor for understanding eruptive styles^5,6^. Understanding these processes is thus important for volcanic hazard forecasting and mitigation. Moreover, the global composition of volcanic rocks also give clues to the formation and evolution of the continental crust^7^. Information regarding volcanoes, their eruptive histories, and their rock compositions can be found in various global databases, including the following two:

- The Geochemistry of Rocks of the Oceans and Continents (GEOROC^8–18^, https://georoc.eu) database, from the Digital Geochemistry Infrastructure (DIGIS), contains numerous volcanic rock samples, together with their location, material, and chemical rock composition. It also contains rock names as provided by the authors of the respective publications. The GEOROC database contains information about the geochemistry of volcanic rocks, namely the quantitative chemical composition (e.g., the concentration of oxides, elements, and some isotopes) together with various types of material that were analysed (rock, glass, mineral) of each sample.
- The Volcanoes of the World (VOTW) database of the Smithsonian’s Global Volcanism Program (GVP^19,20^, https://volcano.si.edu/) provides information pertaining to eruptions from Holocene volcanoes. A list of up to 5 major and minor rock names are provided for some volcanoes, although without the chemical composition of the rocks.

The most abundant element in volcanic rocks is silica (expressed as SiO_2_) and together with sodium (Na_2_O) and potassium (K_2_O) are used to name volcanic rocks, glass, and melt inclusions in 15 fields according to Le Bas *et al*.^21,22^ and Le Maitre *et al*.^23^ in a Total-Alkali Silica (TAS) diagram. This classification was originally made using geochemical data of rock samples compiled from the published literature archived in Computer Library of Analysed Igneous Rocks (CLAIR) database^24^.In this data descriptor, we assign rock types (major and minor) of the global Holocene volcanoes, based on the quantified mass (wt%) of the total alkali and silica oxide of the volcanic rock samples from the GEOROC database, without using pre-defined rock names (as assigned by the authors of the respective publication). We use the TAS of Le Bas^21,22^ categorization to assign a rock name to a volcanic GEOROC sample. This requires agglomerating GEOROC sample datasets belonging to the corresponding GVP volcano^25^, which is achieved by matching the names and locations from both databases. This results in a new dataset, which lists volcanoes, five major and minor rock types, with their percentages, following the TAS nomenclature^21–23^ and then translated into the GVP classification^19,20^ (see Table 1). The major and minor rock types are given by material type (whole rock, glass, and melt inclusion), and for all materials combined. Case examples are provided to validate the data. This dataset has an important reuse value, since it provides data at a compact, yet finer granularity compared to what is currently available in the GVP database. We however acknowledge that it remains a temporal snapshot of GVP and GEOROC which are evolving databases. The rock names for major rocks are expected to remain mostly stable over time, whereas minor rock names, which by definition rely on a small abundance, are susceptible to small variations.

**Table 1:** Rock names correspondence.

Code	Rock names based on TAS nomenclature^21–23^	Code	Rock names based on GVP classification^19,20^
1	FOIDITE	F	Foidite
2	PICROBASALT	B	Basalt/Picro-Basalt
3	BASALT	B	Basalt/Picro-Basalt
4	TEPHRITE/BASANITE	X	Trachybasalt/Tephrite Basanite
5	TRACHYBASALT	X	Trachybasalt/Tephrite Basanite
6	BASALTIC ANDESITE	A	Andesite/Basaltic Andesite
7	ANDESITE	A	Andesite/Basaltic Andesite
8	BASALTIC TRACHYANDESITE	Y	Trachyandesite/Basaltic Trachyandesite
9	TRACHYANDESITE	Y	Trachyandesite/Basaltic Trachyandesite
10	DACITE	D	Dacite
11	RHYOLITE	R	Rhyolite
12	TRACHYTE/TRACHYDACITE	T	Trachyte/Trachydacite
13	PHONO-TEPHRITE	Z	Phono-tephrite/Tephri-phonolite
14	TEPHRI-PHONOLITE	Z	Phono-tephrite/Tephri-phonolite
15	PHONOLITE	P	Phonolite

## Methods

### The GEOROC database

We downloaded in June 2021 the GEOROC precompiled files from the Geochemistry of Rocks of the Oceans and Continents (GEOROC^8–18^, https://georoc.eu of the Digital Geochemistry Infrastructure (DIGIS)). The files used are available in Dataverse “GEOROC data from 2021”^26^. The GEOROC database has changed since then, and is currently delivering updates every three months which are associated with a DOI. The precompiled files are grouped in 11 folders, mainly according to their tectonic settings: Archean cratons, complex volcanic settings, continental flood basalts, convergent margins, intraplate volcanics, ocean basin flood basalts, ocean island groups, oceanic plateaus, rift volcanics, seamounts and submarine ridges. Melt inclusions are stored in a separate folder. In this paper we only use volcanic rock samples: there are ~380,000 in total, out of which more than 138,000 are relevant for Holocene volcanoes. In Dataverse “GEOROC data from 2021”^26^, we also added one folder for complementing the data for three volcanoes, Fuji, Merapi and Rinjani, which we compiled and is not part of the GEOROC dataset. We note that on top of being a temporal snapshot, GEOROC data may not necessarily be representative of the actual rock compositions of the volcanoes, as samples may have been collected because they were out of the ordinary, which may lead to a sampling bias. One suggestion to mitigate these biases is to consider linking the samples with mapped geological units, and thus estimate the abundances of different rock types more accurately. However, implementing such an approach would be a substantial undertaking. It would require extensive fieldwork, detailed geological mapping, and careful sample collection and analysis. This complex and time-consuming project may not be feasible for all volcanic areas or studies, considering the resources and efforts involved.

### Matching sample locations with volcano locations

The GVP lists volcanoes with their name and geographical location (latitude, longitude, country, region, and subregion), but in the GEOROC database, a sample has a LOCATION field, a LOCATION COMMENT field, a range of latitudes (LATITUDE MIN, LATITUDE MAX) and a range of longitudes (LONGITUDE MIN, LONGITUDE MAX). The LOCATION field is of the form REGION/SUBREGION/SUBSUBREGION. To match GVP volcanoes and GEOROC samples, we first match their latitudes/longitudes, and then refine by names. Volcano names in the GEOROC database are typically found inside the LOCATION field, but sometimes inside the LOCATION comment. Based on the 2021 data, 1416 Holocene volcanoes listed by GVP had major rock data, which, after matching with GEOROC data, yield 1044 volcanoes with major rock data as per GEOROC^25^. Note that the original GVP data we used in the paper corresponds to the 2021 release.

### Computing rock names from chemical compositions

**Fig. 1:**
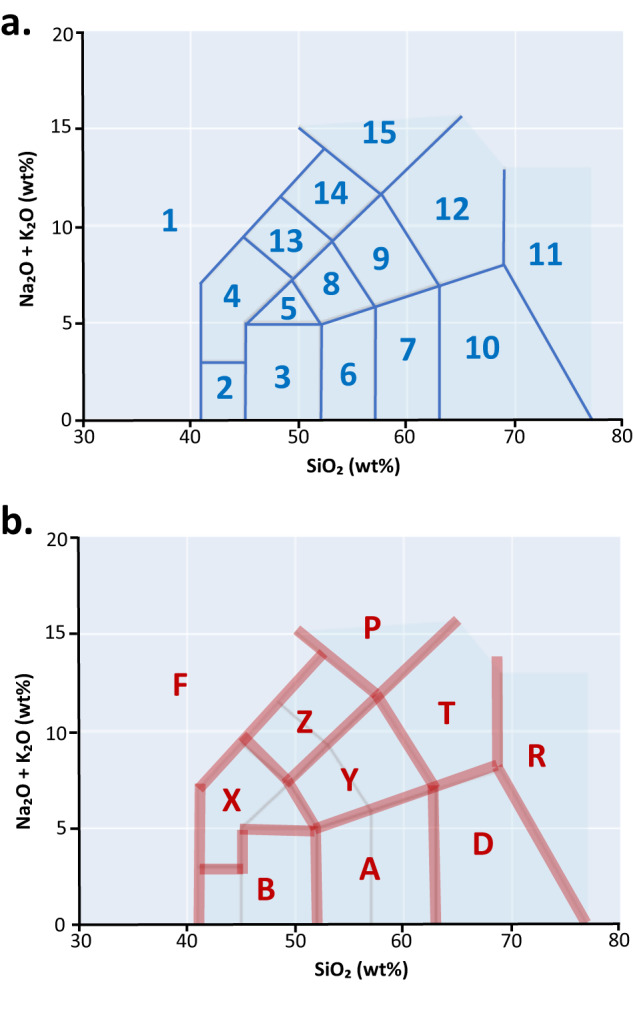
(**a**) The Total Alkali Silica (TAS) diagram described in Le Bas *et al*.^21,22^ and Le Maitre *et al*.^23^ with the 15 field names used to classify the GEOROC volcanic rocks in this paper, which are the following: (1) FOIDITE, (2) PICROBASALT, (3) BASALT, (4) TEPHRITE/BASANITE (5) TRACHYBASALT, (6) BASALTIC ANDESITE, (7) ANDESITE, (8) BASALTIC TRACHYANDESITE, (9) TRACHYANDESITE, (10) DACITE, (11) RHYOLITE, (12) TRACHYTE/TRACHYDACITE, (13) PHONO-TEPHRITE, (14) TEPHRI-PHONOLITE, (15) PHONOLITE. (**b**) The same TAS diagram with with the corresponding rock types based on GVP categorisation.

The rock classification diagram following the TAS scheme^21–23^, was used to identify the rock names, which rely on three main oxide values in volcanic rocks: silica (SiO_2_) together with sodium (Na_2_O) and potassium (K_2_O). The 15 rock names following the TAS fields (Fig. 1a) are the following: 1) FOIDITE, 2) PICROBASALT, 3) BASALT, 4) TEPHRITE/BASANITE 5) TRACHYBASALT, 6) BASALTIC ANDESITE, 7) ANDESITE, 8) BASALTIC TRACHYANDESITE, 9) TRACHYANDESITE, 10) DACITE, 11) RHYOLITE, 12) TRACHYTE/TRACHYDACITE, 13) PHONO-TEPHRITE, 14) TEPHRI-PHONOLITE, 15) PHONOLITE. Using the fields SIO2(WT%), NA2O(WT%) and K2O(WT%) from GEOROC, we computed for each sample a point on the TAS diagram, corresponding to a rock name, in the above list.

To ensure the rock name computation is accurate, one needs to account for the iron concentration, reported as FeO and Fe_2_O_3_. GEOROC contains data fields for FE2O3(WT%), FEO(WT%), and FEOT(WT%). For samples where only FEOT(WT%) is reported we used this value. For samples where FE2O3(WT%) and FEO(WT%) are reported, we recalculated Fe_2_O_3_ into FeO and added it to the reported FEO(WT%), so that for all samples we report FEOT(wt%) as: $\frac{{\rm{FE}}2{\rm{O}}3\left({\rm{WT \% }}\right)}{1.111}+{\rm{FEO}}\left({\rm{WT \% }}\right)$. Moreover, we normalized SIO2(WT%), NA2O(WT%), K2O(WT%) and all other oxides to 100(wt%). We only compute a rock name for samples such that 35 < SIO2(WT%) < 80, FEOT(WT%) > 0, NA2O(WT%) > 0 and K2O(WT%) > 0, which is done after normalization.

We do not exclude any sample compositions if their totals are too high/low. We do not filter samples that are altered either. We note that >95% of the volatiles are water and most of them (including water) are lost to the atmosphere during eruption. Thus, the concentration of remaining volatiles in the rock is <1% and does not have an influence on the normalization. We would also like to add that it is a standard procedure of the community to report normalized oxide compositions to 100% on an anhydrous basis to be able to compare between samples.

### Materials

Each GEOROC volcanic rock sample can represent one out of four types of material analysed: whole rock (WR), volcanic glass (GL), melt inclusion (INC) and mineral/ component including groundmass (MIN) according to the field MATERIAL. Noting that GVP describe their major rocks based on whole rock analysis^19,20^, we thus compute GEOROC major and minor rocks for each material separately, namely for whole rock, volcanic glass and melt inclusions. Using only whole rock (WR) provides a comparison with GVP records. The other records complement the understanding of volcanic rocks and are useful to compare between different types of material analyzed, e.g., melt inclusions compared to whole rock samples.

### Major and minor rocks

For each GVP Holocene volcano, we matched the corresponding GEOROC samples. Each match is a volcanic rock sample of a specific material and is assigned with a major/major rock name, following the described procedure above. The assignment of major rock type is done by number of occurrences in each TAS field (Fig. 1a, Table 1). The most common rock type among all samples from a given volcano becomes its major rock 1, then followed sequentially by major rock 2 to 5 based on the order of abundance. Following GVP rock-type assignment^19,20^, major rock types are those that consist of more than 10% of the total population, when known and quantified. Those that are less then 10% are labelled as minor rock types. We note that when two rock names appear the same number of times, one of the two is chosen arbitrarily over the other, but the user can see the percentages and evaluate.

### Rock names

The dominant rock names for Holocene volcanoes provided by GVP includes the five most common ones, listed in order of decreasing abundance (Fig. 1b, Table 1). The GVP classification is different to the general TAS scheme (Fig. 1a, Table 1) described by Le Bas *et al*.^21,22^ and Le Maitre *et al*.^23^. To be able to compare the major rock names obtained from the GEOROC database to the GVP major rock names, we thus translated the TAS nomenclature to the GVP classification. A correspondence between the names is given in Table 1.

## Data Records

We provide two data files, available in Dataverse “Major rocks from the GEOROC database”^27^. The first row of each file contains the headers, which are the same for both files, and are: Volcano Number, Volcano Name, material, major rock 1, % of major rock 1,major rock 2, % of major rock 2,major rock 3, % of major rock 3, major rock 4, % of major rock 4, major rock 5, % of major rock 5, minor rock 1, % of minor rock 1, minor rock 2, % of minor rock 2, minor rock 3, % of minor rock 3, minor rock 4, % of minor rock 4, minor rock 5, % of minor rock 5.

The rock names in the file “completeGEOROCmajorminorrocks2021.csv”were computed from the GEOROC data based on TAS nomenclature^21–23^. In the file “completeGEOROCmajorminorrocks2021_GVPnames.csv”, the rocks are named according to GVP rock name convention^19,20^. For both data files, the first (empty) field before the first comma stores the row number (which starts from 0 and increases, before ordering the data by volcano number.). A description of the content of each field is provided in Table 2. When no major or minor rock is available, ‘No Data’ is indicated.

**Table 2:** Dataset fields and content.

Field	Content
Volcano Number	GVP Volcano Number
Volcano Name	GVP Volcano Name
material	WR = whole rock, GL = volcanic glass, INC = melt inclusion
major rock 1 to major rock 5	Major rocks are those with most abundance, consisting of more than 10% of the total population, which were computed from the GEOROC data, based on either the TAS nomenclature^21–23^, see Fig. 1a and Table 1, or using the GVP rocks name convention^19,20^, see Fig. 1b and Table 1.
minor rock 1 to minor rock 5	Minor rocks are those consisting of less than 10% of the total population, which were computed from the GEOROC data, based on either the TAS nomenclature Fig. 1a and Table 1, or using the GVP rocks name convention^19,20^, see Fig. 1b and Table 1.

The datasets described in this paper are available in open access repositories:

- The new dataset is available in two CSV files called “Major rocks from the GEOROC database” in the Dataverse repository^27^ DR-NTU (Data), institutional repository of Nanyang Technological University (10.21979/N9/BDRRSI)
- We used two input datasets: 1) The precompiled files of the GEOROC datasets, downloaded in June 2021^26^, can be accessed and downloaded from the Dataverse DR-NTU (Data), institutional repository of Nanyang Technological University repository (10.21979/N9/BJENCK). The original data was downloaded from the GEOROC database (GEOROC^8–18^, https://georoc.eu). We provide this dataset for the purpose of reproducibility of our results, since the GEOROC database provides updated data. Since November 2021, GEOROC uses a new data management system, and the data is updated every 3 months. The record we provide predates the new data management system. This repository also contains 3 datasets that we compiled, with data for the volcanoes Fuji, Merapi and Rinjani 2) The the Global Volcanism Program (GVP) volcano data, downloaded in 2021, can be access and downloaded from Github link (https://github.com/feog/DashVolcano/blob/main/GVP_Volcano_List.xlsx).

## Technical Validation

**Fig. 2:**
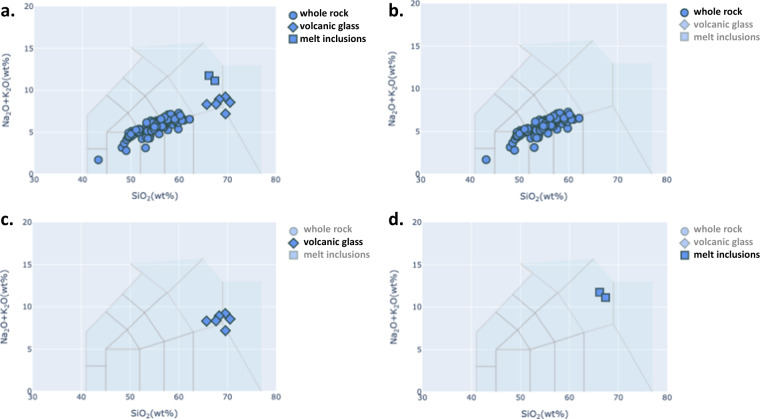
TAS diagram for Kilauea volcano, Hawaii. There are 4722 GEOROC volcanic rock samples, categorised into 3 different types of material analysed (2334 whole rocks, 1238 volcanic glasses and 1150 melt inclusions).TAS plot for different materials and their respective ordering of their 5 major rock types are presented: (**a**) All available type of materials, (**b**) only from whole rock analyses [Basalt (83.7%), Picrobasalt (12.0%), No Data, No Data, No Data], (**c**) only for volcanic glasses [Basalt (74.1%), Basaltic Andesite (17.0%), No Data, No Data, No Data], and (**d**) only for melt inclusions [Basalt (75.7%), Basaltic Andesite (21.2%), No Data, No Data, No Data].

**Fig. 3:**
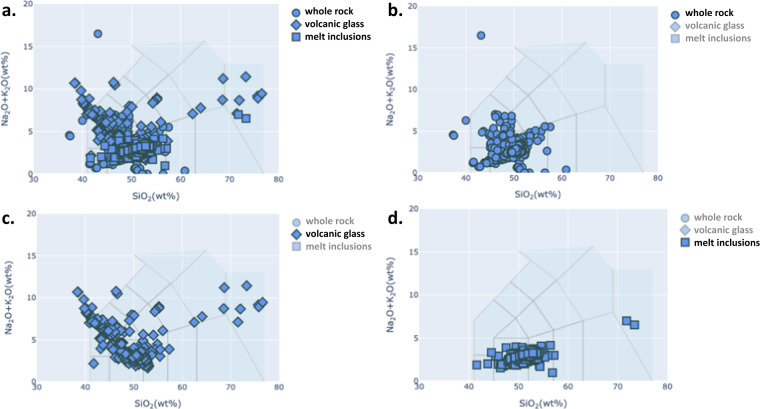
TAS diagram for Taranaki volcano, New Zealand. There are 145 GEROC volcanic rock samples, categorised into 3 different types of material analysed (137 whole rocks, 6 volcanic glasses and 2 melt inclusions). TAS plot for different materials and their respective ordering of their 5 major rock types are presented: (**a**) All available type of materials, (**b**) only from whole rock analyses [Basaltic Trachyandesite (38.7%), Basalt (17.5%), Trachybasalt (12.4%), Basaltic Andesite (11.7%), Trachyandesite (11.7%)], (**c**) only for volcanic glasses [Rhyolite (83.3%), Trachyte/Trachydacite (16.7%), No Data, No Data, No Data], and (**d**) only for melt inclusions [Trachyte/Trachydacite (100.0%), No Data, No Data, No Data, No Data].

To validate the data, we first visually compared the TAS diagram obtained from GEOROC data samples with the five major rock types that were computed and stored in the new dataset. In Fig. 2, four TAS diagrams are shown for Kilauea volcano: one for whole rock samples, one for volcanic glass samples, one for melt inclusion samples, and one for all samples combined. We can compare their relative abundances on the TAS diagrams, and their major rock names. A similar visual comparison is provided for the Taranaki volcano (New Zealand) on Fig. 3. This volcano has fewer samples than Kilauea. Detail explanation on the assignments of major and minor rock types with case example of the Taranaki volcano can be seen in the appendix (supplementary information).

**Fig. 4:**
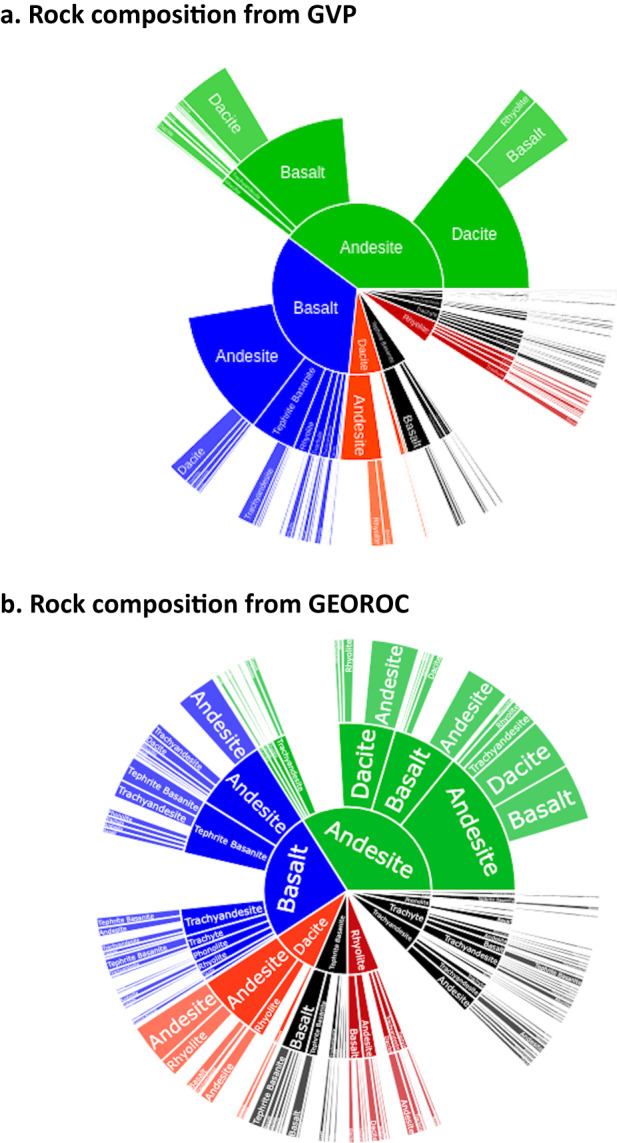
Overall presentation of the GEOROC volcanic rock’s chemical composition of the global Holocene volcanoes plotted in sunburst diagram showing the rock names, with major rock 1 at the inner circle, followed by major rock 2 in the middle, and major rock 3 in the outer circle. To illustrate the comparison with GVP major rocks, we translated the 15 TAS classifications of the GEOROC following the GVP nomenclature^20^. Both GEOROC and GVP are based on whole rocks analyses. (**a**) The GVP major rock types covering 1416 Holocene volcanoes, and (**b**) The GEOROC major rock types covering 1044 volcanoes.

To validate the soundness of our dataset, we provide an overall comparison with major rock types that are available in the GVP database, using only whole rock samples. Major Rock 1 for Kilauea volcano is Basalt/Picro-Basalt (GVP^19^, https://volcano.si.edu/volcano.cfm?vn=332010). For Taranaki volcano, the Major Rock 1 is Andesite/Basaltic Andesite (GVP^19^, https://volcano.si.edu/volcano.cfm?vn=241030). We do not expect to find the exact same result, since different compositional datasets are being used by GVP and this paper. GVP estimated rock types for each volcano based upon geological, geochemical and/or petrological studies published prior to 2010, with relative abundances estimated from eruptive histories and precedence given to rock types composing younger edifices^20^. In contrast, our dataset uses published analyses compiled within GEOROC downloaded in June 2021^26^ following the procedures previously described, but does not weight samples according to eruptive history or mapped extent. Nevertheless, we found that the overall distribution of major rock types are similar (Fig. 4), and that the two datasets agree on the distribution of the first major rock among andesite, basalt, dacite and rhyolite.

## Supplementary information

Appendix

## Data Availability

The code used to generate the data descriptor is a minor modification of the function available in the github link (https://github.com/feog/DashVolcano).
