# Supplementary material for: Global volcanic rock classification of Holocene volcanoes
Source: Sci Data. 2023 Jul 1;10:422. doi: 10.1038/s41597-023-02324-7 (PMC10314901; doi:10.1038/s41597-023-02324-7)
Supplement: Supplementary file 1 — Appendix [file 41597_2023_2324_MOESM1_ESM.pdf]

Appendix – Assigning Taranaki volcano rock types

This appendix will provide case example on how the ordering of the 5 major and 5 minor rock types from GEOROC volcanic rock samples of the Taranaki volcano (New Zealand) was assigned, for the 3 different types of material analysed: whole rocks (WR), volcanic glasses (GL) and melt inclusions (INC).

1. The weight percent (wt%) of the SiO_2, Na_2O, and K_2O of the 145 GEOROC matched samples to Taranaki were automatically re-computed [SIO2(WT%), K2O(WT%), NA2O(WT%)], where all data normalized to 100% volatile-free and accounted with its total iron oxide (see detail in Oggier et al.[1]).
2. Then the rock type of each sample was assigned following the TAS classification scheme[2,3,4](Table A-1), considering only samples with 35<SIO2(WT%)<80, FEOT(WT%)>0, NA2O(WT%)>0 and K2O(WT%)>0.

Table A-1
The rock types of the 145 GEROC volcanic rock samples, categorised into 3 different types of material analysed (137 whole rocks, 2 melt inclusions, and 6 volcanic glasses).

	SAMPLE NAME	SIO2(WT%)	K2O(WT%)	NA2O(WT%)	MATERIAL	ROCK TYPE
1	s_UL2 [20347]	43.28217977	0.27142708	1.419772419	WR	PICROBASALT
2	s_T95-3X2 [20347]	42.41695272	0.461439653	2.316072105	WR	PICROBASALT
3	s_KW4_P [21470]	56.53143112	2.039679001	3.901025412	WR	BASALTIC TRACHYANDESITE
4	s_KW4_DC [21470]	57.34486267	2.522889115	4.028484232	WR	TRACHYANDESITE
5	s_KW7T [21470]	56.87744459	2.009995654	3.87874837	WR	BASALTIC TRACHYANDESITE
6	s_KW7M [21470]	57.05199732	1.896898014	3.838428922	WR	ANDESITE
7	s_KW7B [21470]	59.82447083	1.806917914	3.572534848	WR	ANDESITE
8	s_UIG1 [21470]	59.55391099	2.405180389	4.265597698	WR	TRACHYANDESITE
9	s_UIG2 [21470]	58.80670208	2.247650184	4.209235799	WR	TRACHYANDESITE
10	s_UIG3 [21470]	58.51577757	2.00431699	4.265597698	WR	TRACHYANDESITE
11	s_UIG4 [21470]	60.51882316	2.383212064	4.291890752	WR	TRACHYANDESITE
12	s_UIG5T [21470]	61.02515525	2.399747395	4.020629407	WR	ANDESITE
13	s_UIG5B [21470]	60.45459329	2.388719352	4.272335052	WR	TRACHYANDESITE
14	s_UIG6T [21470]	58.62510675	1.868061486	4.056362084	WR	ANDESITE
15	s_UIG6B [21470]	60.46586174	2.436157709	4.284645795	WR	TRACHYANDESITE
16	s_UIG7_P [21470]	60.64446831	2.352309345	4.285714286	WR	TRACHYANDESITE
17	s_UIG7_DC [21470]	60.28775531	2.444913975	4.326390985	WR	TRACHYANDESITE
18	s_MD1 [21470]	50.17054264	1.757105943	3.369509044	WR	TRACHYBASALT
19	s_MD1T_DC1 [21470]	49.97454434	1.618979737	3.115772325	WR	BASALT
20	s_MD1T_V1 [21470]	49.93875051	1.602694978	3.123723969	WR	BASALT
21	s_MD1T_V2 [21470]	50.11432135	1.47578466	3.013926419	WR	BASALT
22	s_MD1T_DC2 [21470]	51.76458691	1.7393063	3.316816665	WR	TRACHYBASALT
23	s_MD2 [21470]	51.54755784	1.686375321	3.259640103	WR	BASALT
24	s_MD3B_V [21470]	50.7610037	1.614561909	3.146853147	WR	BASALT

	SAMPLE NAME	SIO2(WT%)	K2O(WT%)	NA2O(WT%)	MATERIAL	ROCK TYPE
25	s_MD3B_DC [21470]	51.18030118	1.72975173	3.266178266	WR	BASALT
26	s_MD3M [21470]	51.13122172	1.683678838	3.314742713	WR	BASALT
27	s_MD3T_V [21470]	49.35320294	1.376384146	2.825209562	WR	BASALT
28	s_MD3T_DC1 [21470]	50.75953291	1.746409011	3.337811305	WR	TRACHYBASALT
29	s_MD3T_DC2 [21470]	51.6031152	1.770001011	3.256801861	WR	TRACHYBASALT
30	s_MA [21470]	51.85803758	1.743215031	3.402922756	WR	BASALTIC TRACHYANDESITE
31	s_MC [21470]	48.64063778	1.226492232	2.422322159	WR	BASALT
32	s_89/74 [6624]	48.49673414	1.711430587	3.162426085	WR	BASALT
33	s_89/75 [6624]	47.37569978	1.564161772	3.183529253	WR	BASALT
34	s_89/76 [6624]	50.59375438	2.270713754	3.659414727	WR	TRACHYBASALT
35	s_89/77 [6624]	45.15193871	1.394478527	2.725157383	WR	BASALT
36	s_89/81 [6624]	51.01555653	2.337161285	3.449650057	WR	TRACHYBASALT
37	s_89/82 [6624]	53.64333265	2.668494136	3.734005929	WR	BASALTIC TRACHYANDESITE
38	s_89/85 [6624]	52.09842976	1.867697266	3.425675889	WR	BASALTIC TRACHYANDESITE
39	s_T10 [6624]	48.60014633	0.617560581	2.236317861	WR	BASALT
40	s_T15 [6624]	45.43705301	0.252844277	2.331786109	WR	BASALT
41	s_T89/10 [6685]	50.59375438	2.270713754	3.659414727	WR	TRACHYBASALT
42	s_T89/15 [6685]	51.01555653	2.337161285	3.449650057	WR	TRACHYBASALT
43	s_T89/13 [6685]	51.04616051	2.347057389	3.188631752	WR	TRACHYBASALT
44	s_T89/19 [6685]	52.81977729	2.545300862	3.550024887	WR	BASALTIC TRACHYANDESITE
45	s_T90/2B [6685]	54.81313917	2.864367168	3.511466728	WR	BASALTIC TRACHYANDESITE
46	s_T89/18BB [6685]	55.68975002	2.953171916	3.698632788	WR	BASALTIC TRACHYANDESITE
47	s_T89/24 [6685]	50.7673788	2.060394533	3.619612017	WR	TRACHYBASALT
48	s_T90/21 [6685]	51.52428102	2.097848214	3.461919922	WR	BASALTIC TRACHYANDESITE
49	s_T89/9 [6685]	47.37569978	1.564161772	3.183529253	WR	BASALT
50	s_T89/8 [6685]	48.49673414	1.711430587	3.162426085	WR	BASALT
51	s_T90/10 [6685]	49.80047673	1.928662055	3.028092598	WR	BASALT
52	s_T90/4D [6685]	50.10958162	1.599340887	3.040607383	WR	BASALT
53	s_T89/22 [6685]	51.41428686	1.93258714	3.87451045	WR	BASALTIC TRACHYANDESITE
54	s_T89/21 [6685]	52.09842976	1.867697266	3.425675889	WR	BASALTIC TRACHYANDESITE
55	s_BR-6 [6685]	52.58744408	2.075325918	3.361840175	WR	BASALTIC TRACHYANDESITE
56	s_T90/32A [6685]	47.49183412	1.74493714	2.862066209	WR	BASALT
57	s_T90/45A [6685]	52.38152206	1.916282029	3.426652101	WR	BASALTIC TRACHYANDESITE
58	s_T90/46 [6685]	52.12730394	1.896904233	3.549652475	WR	BASALTIC TRACHYANDESITE
59	s_T90/32C [6685]	53.48620705	1.922397314	3.787975003	WR	BASALTIC TRACHYANDESITE
60	s_T90/42A [6685]	44.71073943	1.096747492	2.623054419	WR	TEPHRITE/BASANITE
61	s_T90/42C [6685]	52.18664124	1.480339195	2.988786096	WR	BASALTIC ANDESITE
62	s_T89/33A [6685]	51.95327178	2.043318774	3.717704991	WR	BASALTIC TRACHYANDESITE
63	s_T89/36 [6685]	55.1103624	2.077096192	3.788777314	WR	BASALTIC TRACHYANDESITE
64	s_T90/41 [6685]	54.14640028	1.673274175	3.2619446	WR	BASALTIC ANDESITE
65	s_T89/6A [6685]	52.65501776	1.063245079	3.433598787	WR	BASALTIC ANDESITE
66	s_T89/3 [6685]	57.07523821	1.733931287	4.16143509	WR	ANDESITE
67	s_AZ06-73 [15820]	49.72544234	1.281269067	3.640431157	WR	BASALT
68	s_AZ06-68 [15820]	52.62997253	1.180181097	3.042018517	WR	BASALTIC ANDESITE

	SAMPLE NAME	SIO2(WT%)	K2O(WT%)	NA2O(WT%)	MATERIAL	ROCK TYPE
69	s_AZ04-30 [15820]	56.97275479	1.826437941	3.864783047	WR	BASALTIC ANDESITE
70	s_AZ04-20 [15820]	53.40874659	1.474598525	3.545096455	WR	BASALTIC ANDESITE
71	s_AZ04-6 [15820]	49.44105691	1.280487805	3.119918699	WR	BASALT
72	s_AZ04-1 [15820]	59.8344743	1.941350771	4.209665883	WR	ANDESITE
73	s_AZ06-32 [15820]	56.11342616	1.809126944	4.177253786	WR	BASALTIC TRACHYANDESITE
74	s_AZ06-12 [15820]	55.01572168	2.038746323	3.986205498	WR	BASALTIC TRACHYANDESITE
75	s_AZ06-01 [15820]	60.61251394	2.301997769	4.107088531	WR	ANDESITE
76	s_AZ05-22 [15820]	55.62154001	2.174131857	3.895319577	WR	BASALTIC TRACHYANDESITE
77	s_AZ05-21 [15820]	54.41326012	1.738865162	3.630262355	WR	BASALTIC ANDESITE
78	s_AZ06-93 [15820]	51.98648372	1.812410403	3.594101986	WR	BASALTIC TRACHYANDESITE
79	s_AZ06-83 [15820]	62.52187227	2.230971129	4.352580927	WR	ANDESITE
80	s_AZ05-55 [15820]	52.88548696	1.722227818	3.504884681	WR	BASALTIC TRACHYANDESITE
81	s_AZ05-33 [15820]	54.80991072	1.976125991	3.841909921	WR	BASALTIC TRACHYANDESITE
82	s_AZ06-51 [15820]	59.23418872	2.323701758	4.041220449	WR	TRACHYANDESITE
83	s_T90/39 [9706]	53.24862938	2.020196809	3.213522366	WR	BASALTIC TRACHYANDESITE
84	s_BR1 [9706]	53.56806686	2.009987641	3.507997299	WR	BASALTIC TRACHYANDESITE
85	s_T90/2B [9706]	54.81313917	2.864367168	3.511466728	WR	BASALTIC TRACHYANDESITE
86	s_T89/13 [9706]	49.36369337	1.852069189	3.434238848	WR	TRACHYBASALT
87	s_T89/15 [9706]	51.01555653	2.337161285	3.449650057	WR	TRACHYBASALT
88	s_T90/16 [9706]	51.31443644	2.099226945	3.228144547	WR	TRACHYBASALT
89	s_T89/12A [9706]	52.76725718	2.222969064	3.607615048	WR	BASALTIC TRACHYANDESITE
90	s_T90/13 [9706]	51.04616051	2.347057389	3.188631752	WR	TRACHYBASALT
91	s_T90/3 [9706]	54.47488098	2.641837614	3.46563644	WR	BASALTIC TRACHYANDESITE
92	s_SD20 [9959]	53.45923492	2.607532877	3.386906172	WR	BASALTIC TRACHYANDESITE
93	s_SD35 [9959]	53.47180039	2.58796587	3.550034967	WR	BASALTIC TRACHYANDESITE
94	s_TO4-22 [9959]	53.61332491	2.594501973	3.436997079	WR	BASALTIC TRACHYANDESITE
95	s_TO4-96 [9959]	52.7929021	2.510325316	3.613347046	WR	BASALTIC TRACHYANDESITE
96	s_P10 [9959]	52.98667111	2.501435021	3.414506359	WR	BASALTIC TRACHYANDESITE
97	s_SD24 [9959]	53.63701704	2.633789009	3.556576399	WR	BASALTIC TRACHYANDESITE
98	s_TO4-19 [9959]	53.58991785	2.558288587	3.391888239	WR	BASALTIC TRACHYANDESITE
99	s_TO4-65 [9959]	53.53409267	2.599953246	3.444218507	WR	BASALTIC TRACHYANDESITE
100	s_SD9A [9959]	53.52268054	2.674237405	3.660480986	WR	BASALTIC TRACHYANDESITE
101	s_SD9C [9959]	54.63078143	2.90921941	3.781037605	WR	BASALTIC TRACHYANDESITE
102	s_SD28 [9959]	54.19789838	2.860997258	3.760979839	WR	BASALTIC TRACHYANDESITE
103	s_TO4-1 [9959]	54.51226976	2.859101332	3.767954735	WR	BASALTIC TRACHYANDESITE
104	s_TO4-21 [9959]	53.79151716	2.735482748	3.729343262	WR	BASALTIC TRACHYANDESITE
105	s_TO4-24 [9959]	56.54020463	3.087811545	3.781381523	WR	TRACHYANDESITE
106	s_SD34 [9959]	53.9645205	2.856666992	3.774205728	WR	BASALTIC TRACHYANDESITE
107	s_SD39 [9959]	54.6056175	2.927838939	3.771132355	WR	BASALTIC TRACHYANDESITE
108	s_SD9B [9959]	52.99124344	2.542365421	3.528954987	WR	BASALTIC TRACHYANDESITE
109	s_SD27 [9959]	53.20532956	2.567549416	3.537512529	WR	BASALTIC TRACHYANDESITE
110	s_SD29 [9959]	53.10098177	2.533607381	3.543240398	WR	BASALTIC TRACHYANDESITE
111	s_SD33 [9959]	53.15272782	2.538779665	3.518159087	WR	BASALTIC TRACHYANDESITE
112	s_37944 [9777]	54.54640033	1.009155223	3.308364544	WR	BASALTIC ANDESITE

	SAMPLE NAME	SIO2(WT%)	K2O(WT%)	NA2O(WT%)	MATERIAL	ROCK TYPE
113	s_37947 [9777]	53.34757835	1.658526659	3.530728531	WR	BASALTIC ANDESITE
114	s_37946 [9777]	53.3984534	1.210826211	3.164428164	WR	BASALTIC ANDESITE
115	s_37951 [9777]	53.58163265	1.846938776	3.295918367	WR	BASALTIC ANDESITE
116	s_37948 [9777]	53.84694086	1.227747084	3.018211582	WR	BASALTIC ANDESITE
117	s_37949 [9777]	54.92254912	1.798523755	3.472294417	WR	BASALTIC ANDESITE
118	s_37950 [9777]	56.94284819	2.350851531	3.928534113	WR	TRACHYANDESITE
119	s_37945 [9777]	56.27430911	2.067553736	3.817809621	WR	BASALTIC TRACHYANDESITE
120	s_37953 [9777]	56.79951323	2.231011054	3.50877193	WR	BASALTIC ANDESITE
121	s_37954 [9777]	57.93384224	2.921119593	3.623409669	WR	TRACHYANDESITE
122	s_37952 [9777]	59.79125762	3.058799215	3.596155833	WR	TRACHYANDESITE
123	s_37955 [9777]	60.30666125	3.188464663	3.818034119	WR	TRACHYANDESITE
124	s_T89/16 [9706]	53.64333265	2.668494136	3.734005929	WR	BASALTIC TRACHYANDESITE
125	s_T90/28 [6685]	50.84302002	2.105591663	3.612259475	WR	TRACHYBASALT
126	s_T89/11 [9706]	45.15193871	1.394478527	2.725157383	WR	BASALT
127	s_T89/14 [9706]	45.53888719	1.422805207	2.918574785	WR	BASALT
128	s_T90/27 [6685]	49.10990185	2.013533685	3.648375255	WR	TRACHYBASALT
129	s_T90/4A [6685]	50.35183263	1.643724525	3.111004045	WR	BASALT
130	s_EB-4B [12805]	51.73352147	1.884700665	3.446885709	WR	BASALTIC TRACHYANDESITE
131	s_E03-61B [12805]	51.0993404	1.858884669	3.41794923	WR	TRACHYBASALT
132	s_EB-24C [12805]	55.21391727	2.194801254	3.347830484	WR	BASALTIC ANDESITE
133	s_E03-19 [12805]	55.23953606	2.218860313	3.691376702	WR	BASALTIC TRACHYANDESITE
134	s_EB-4P [12805]	54.80012269	2.126571925	3.220529598	WR	BASALTIC ANDESITE
135	s_E03-50 [12805]	55.30624621	2.365069739	3.305033354	WR	BASALTIC TRACHYANDESITE
136	s_E03-57 [12805]	54.91048852	2.336401335	3.297259027	WR	BASALTIC TRACHYANDESITE
137	s_EB-26 [12805]	56.23672231	2.731411229	3.834092059	WR	TRACHYANDESITE
138	samp. T90/27	66.23961565	6.445801221	5.314783305	INC	TRACHYTE/TRACHYDACITE
139	samp. T90/28	67.50801282	5.46875	5.679086538	INC	TRACHYTE/TRACHYDACITE
140	s_EG-2 [9776]	70.86756161	4.88879984	4.478060509	GL	RHYOLITE
141	s_EG-6 [9776]	67.79134962	4.175010012	4.395274329	GL	TRACHYTE/TRACHYDACITE
142	s_EG-7 [9776]	70.37667802	4.858745742	4.327790022	GL	RHYOLITE
143	s_EG-11 [9776]	70.18664537	4.780916259	3.882623016	GL	RHYOLITE
144	s_EG-12 [9776]	71.94352658	3.844998498	3.584660058	GL	RHYOLITE
145	s_EGMONT [10219]	70.64456343	4.468698517	4.098023064	GL	RHYOLITE

3. The assignment of major rock type is done by number of occurrences in each TAS field (Table A-2). The most common rock type among all samples from a given volcano becomes its major rock 1, then followed sequentially by major rock 2 to 5 based on the order of abundance. Major rock types (Table A-3) are those that consist of more than 10% of the total population, when known and quantified. Those that are less then 10% are labelled as minor rock types (Table A-4).

Table A-2

Statistics of the Taranaki rock types.

Material type	Rock types	Counts	Percentage
Whole rock (WR)	BASALTIC TRACHYANDESITE	53	38.7
	BASALT	24	17.5
	TRACHYBASALT	17	12.4
	BASALTIC ANDESITE	16	11.7
	TRACHYANDESITE	16	11.7
	ANDESITE	8	5.8
	PICROBASALT	2	1.5
	TEPHRITE/BASANITE	1	0.7
Melt inclusions (INC)	TRACHYTE/TRACHYDACITE	2	100
Volcanic glasses (GL)	RHYOLITE	5	83.3
	TRACHYTE/TRACHYDACITE	1	16.7

Table A-3

The assigned 5 major rock types of Taranaki volcano, following TAS nomenclature[2,3,4]

Material	Major rock1	%	Major rock2	%	Major rock3	%	Major rock4	%	Major rock5	%
WR	BASALTIC TRACHYANDESITE	38.7	BASALT	17.5	TRACHYBASALT	12.4	BASALTIC ANDESITE	11.7	TRACHY ANDESITE	11.7
INC	TRACHYTE/ TRACHYDACITE	100	No Data	No Data	No Data	No Data	No Data	No Data	No Data	No Data
GL	RHYOLITE	83.3	TRACHYTE/ TRACHYDACITE	16.7	No Data	No Data	No Data	No Data	No Data	No Data

Table A-4

The assigned 5 minor rock types of Taranaki volcano, following TAS nomenclature[2,3,4]

Material	Minor rock1	%	Minor rock2	%	Minor rock3	%	Minor rock4	%	Minor rock5	%
WR	ANDESITE	5.8	PICROBASALT	1.5	TEPHRITE/ BASANITE	0.7	No Data	No Data	No Data	No Data
INC	No Data	No Data	No Data	No Data	No Data	No Data	No Data	No Data	No Data	No Data
GL	No Data	No Data	No Data	No Data	No Data	No Data	No Data	No Data	No Data	No Data

4. The 5 major (Table A-5) and 5 minor (Table A-6) rock types then translated into the GVP classification[5,6].

Table A-5

The assigned 5 major rock types of Taranaki volcano, following GVP classification[5,6]

Material	Major rock1	%	Major rock2	%	Major rock3	%	Major rock4	%	Major rock5	%
WR	Trachyandesite / Basaltic Trachyandesite	38.7	Basalt / Picro-Basalt	17.5	Trachybasalt / Tephrite Basanite	12.4	Andesite / Basaltic Andesite	11.7	Trachyandesite / Basaltic Trachyandesite	11.7
INC	Trachyte / Trachydacite	100	No Data	No Data	No Data	No Data	No Data	No Data	No Data	No Data
GL	Rhyolite	83.3	Trachyte / Trachydacite	16.7	No Data	No Data	No Data	No Data	No Data	No Data

Table A-6

The assigned 5 minor rock types of Taranaki volcano, following GVP classification[5,6]

Material	Minor rock1	%	Minor rock2	%	Minor rock3	%	Minor rock4	%	Minor rock5	%
WR	Andesite / Basaltic Andesite	5.8	Basalt / Picro-Basalt	1.5	Trachybasalt / Tephrite Basanite	0.7	No Data	No Data	No Data	No Data
INC	No Data	No Data	No Data	No Data	No Data	No Data	No Data	No Data	No Data	No Data
GL	No Data	No Data	No Data	No Data	No Data	No Data	No Data	No Data	No Data	No Data

References:

1. Oggier, F., Widiwijayanti, C. & Costa, F., Integrating global geochemical volcano rock composition with eruption history datasets, Front. Earth Sci., 11, doi: 10.3389/feart.2023.1108056 (2023).

2. Le Bas, M.J., Le Maitre, R.W., Streckeisen, A., Zanettin, B. IUGS Subcommission on the Systematics of Igneous Rocks, A Chemical Classification of Volcanic Rocks Based on the Total Alkali-Silica Diagram, Journal of Petrology, Volume 27, Issue 3, June 1986, Pages 745–750, https://doi.org/10.1093/petrology/27.3.745 (1986).

3. Le Bas, M.J., Le Maitre, R.W. & Woolley, A.R. The construction of the Total Alkali-Silica chemical classification of volcanic rocks. Mineralogy and Petrology 46, 1–22. https://doi.org/10.1007/BF01160698 (1992).

4. Le Maitre, R. W., Streckeisen, A., Zanettin, B., Le Bas, M. J., Bonin, B., and Bateman, P. (Eds.). Igneous rocks: a classification and glossary of terms: recommendations of the International Union of Geological Sciences, Subcommission on the Systematics of Igneous Rocks. Cambridge University Press. https://doi.org/10.1017/CBO9780511535581 (2002).

5. Global Volcanism Program. Volcanoes of the World, v. 4.1.1.0 (24 Feb 2021). Venzke, E (ed.). Smithsonian Institution. Downloaded 24 Feb 2021. https://doi.org/10.5479/si.GVP.VOTW4-2013 (2013).

6. Siebert, L., Simkin, T., Kimberly, P. Volcanoes of the World. 3rd edition. Berkeley: University of California Press (2011).
